# High Avidity CD8^+^ T Cells Efficiently Eliminate Motile HIV-Infected Targets and Execute a Locally Focused Program of Anti-Viral Function

**DOI:** 10.1371/journal.pone.0087873

**Published:** 2014-02-13

**Authors:** Maria Hottelet Foley, Talitha Forcier, Elizabeth McAndrew, Michael Gonzalez, Huabiao Chen, Boris Juelg, Bruce D. Walker, Darrell J. Irvine

**Affiliations:** 1 Department of Biological Engineering, Massachusetts Institute of Technology, Cambridge, Massachusetts, United States of America; 2 Ragon Institute of Massachusetts General Hospital, Massachusetts Institute of Technology, and Harvard University, Cambridge, Massachusetts, United States of America; 3 Koch Institute for Integrative Cancer Research, Massachusetts Institute of Technology, Cambridge, Massachusetts, United States of America; 4 Department of Material Science and Engineering, Massachusetts Institute of Technology, Cambridge, Massachusetts, United States of America; 5 Howard Hughes Medical Institute, Chevy Chase, Maryland, United States of America; La Jolla Institute for Allergy and Immunology, United States of America

## Abstract

The dissemination of HIV from an initial site of infection is facilitated by motile HIV-infected CD4^+^ T-cells. However, the impact of infected target cell migration on antigen recognition by HIV-specific CD8^+^ T-cells is unclear. Using a 3D in vitro model of tissue, we visualized dynamic interactions between HIV-infected or peptide-pulsed CD4^+^ T-cells and HIV-specific CD8^+^ T-cells. CTLs engaged motile HIV-infected targets, but ∼50% of targets broke contact and escaped. In contrast, immobilized target cells were readily killed, indicating target motility directly inhibits CD8^+^ T-cell function. Strong calcium signals occurred in CTLs killing a motile target but calcium signaling was weak or absent in CTLs which permitted target escape. Neutralization of adhesion receptors LFA-1 and CD58 inhibited CD8^+^ T-cell function within the 3D matrix, demonstrating that efficient motile target lysis as dependent on adhesive engagement of targets. Antigen sensitivity (a convolution of antigen density, TCR avidity and CD8 coreceptor binding) is also critical for target recognition. We modulated this parameter (known as functional avidity but referred to here as “avidity” for the sake of simplicity) by exploiting common HIV escape mutations and measured their impact on CTL function at the single-cell level. Targets pulsed with low avidity mutant antigens frequently escaped while CTLs killed targets bearing high avidity antigen with near-perfect efficiency. CTLs engaged, arrested, and killed an initial target bearing high avidity antigen within minutes, but serial killing was surprisingly rare. CD8 cells remained committed to their initial dead target for hours, accumulating TCR signals that sustained secretion of soluble antiviral factors. These data indicate that high-avidity CD8^+^ T-cells execute an antiviral program in the precise location where antigen has been sensed: CTL effector functions are spatiotemporally coordinated with an early lytic phase followed by a sustained stationary secretory phase to control local viral infection.

## Introduction

HIV, the causative agent of an ongoing global epidemic, primarily infects CD4^+^ T lymphocytes [1]. Studies in humanized mice and non-human primate models of HIV have revealed that HIV-infected CD4^+^ T cells are motile and infected cell migration and trafficking contribute to the spread of virus *in vivo* [2], [3]. The outcome of infection depends on a race between expansion of infection versus containment by effective immune responses [2], [4], [5], [6]. HIV-specific CD8^+^ T cells contribute to control of viremia in natural infection and following vaccination, but the characteristics of protective responses are still being defined [7], [8], [9], [10]. The lysis and elimination of HIV-infected targets is dependent on receptor-mediated signaling triggered during physical contact with a target cell [11], [12], [13], [14]. Notably, secretion of soluble anti-viral factors by CTL also contributes to suppression of viral replication *in vitro* [14]. In HIV-and SIV- infected subjects, CTL polyfunctionality (the combination of lytic function and secretion of multiple soluble factors by individual CTL) correlates with long-term control of infection *in vivo* [15], [16]. Furthermore, the potent *ex vivo* ability of CTLs from individuals newly infected with HIV to suppress viral replication has been identified as a powerful predictor of low viral setpoint following acute infection and a delayed progression to disease [17]. Intravital and whole-organ imaging studies in mice have shown that CD8^+^ T cells actively migrate through three-dimensional tissue compartments in search of antigen [18], [19], and the CTL response to non-motile targets has been characterized [20], [21], [22]. However, the effect of target cell migration (a circumstance particularly relevant to the case of HIV infection) on the antiviral function of CTLs has not been determined.

The fundamentals of CTL function have been established in traditional liquid suspension culture, but this *in vitro* approach does not support the physiological migration that occurs within the extracellular matrix (ECM). In the absence of migration, CTL-target contact leads to immunological synapse (IS) formation [13] and cytolytic killing is rapid (∼15 minutes) upon recognition of a few peptide-MHC-I on targets [23]. Such cytotoxic T lymphocytes (CTLs) can kill multiple targets in liquid suspension [24], [25] and it has been proposed that effective CD8^+^ T cells may eliminate viral infection *in vivo* through serial engagement and killing of many targets [25], [26], [27]. However, the response of motile CTL to migrating target cells has not been examined.

While CD8^+^ T cells are equipped with preformed stores of lytic granules for rapid target killing [24], [28], [29] they also produce and secrete anti-viral factors including cytokines and chemokines in an “on-demand” fashion. This response occurs over several hours in liquid suspension cultures and requires sufficiently strong TCR signals for induction of gene transcription and new protein synthesis [30], [31], [32]. How migrating CD8^+^ T cells coordinate these temporally-distinct anti-viral functions for effective protection of infected tissue microenvironments is not known.

Here we utilized a 3D collagen model of peripheral tissue to support the migration of human HIV-specific CD8^+^ T cells and HIV-infected target cells, a matrix that has been shown to reconstitute immune cell engagement dynamics and function strikingly similar to those observed *in vivo* [33], [34], [35]. Using a novel videomicroscopy approach to capture ≥10 continuous hours of cell migration and effector-target engagement dynamics, the histories of individual cells were captured for both effector cells and their targets. These experiments revealed two stages of CD8^+^ T cell dynamics that were coordinated with distinct phases of their effector functions: An early “commitment phase,” marked by engagement and killing of a motile target (∼15 min), was followed by a prolonged “secretory phase” lasting many hours. In the secretory phase, CD8^+^ T cells accumulated TCR signals from their initial killed target, remaining arrested and maintaining contact with the dead target for 3–10 hrs or more; continued TCR signaling during this phase was required for maximal secretion of antiviral cytokines and chemokines. CD8^+^ T cells exhibiting high functional avidity efficiently killed their targets and successfully enter both the commitment and secretory phases. Conversely, CD8^+^ T cells with low avidity not only permitted target escape prompted by target cell migration, but also failed to mount a robust secretory phase. These findings indicate that migration of both target and effector cells impacts CD8^+^ T cell function, with important implications for viral replication and dissemination of HIV, which is predominantly an infection within tissues.

## Results

### In Situ Reporters Enabling Continuous Videomicroscopy of CD8^+^ T Cells and Primary CD4^+^ Target Cells Migrating in ECM

**Figure 1:**
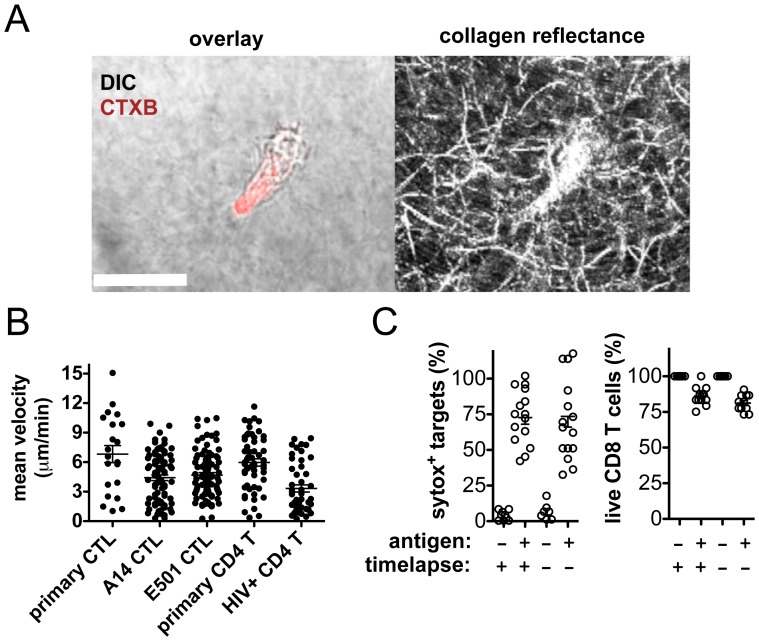
*In situ* reporters enabling continuous videomicroscopy of CTLs and primary CD4^+^ target cells in ECM. (**A**) Confocal images of CTL clone A14 labeled with CTXB (red) migrating through 3D ECM (left: brightfield/CTXB fluorescence overlay, right: reflectance image of collagen type I fibers). Scale bar 20 µm. (**B**) Mean velocities for individual cells tracked for one hour in collagen. HIV-specific CTLs (primary or clones), primary uninfected CD4^+^ T cells or HIV-infected CD4^+^ T cells were cultured independently within ECM. CD4^+^ T cells were infected with HIV-1 NL4-3-GFP [61] and flow-sorted to >95% GFP^+^ populations on day 3 post-infection. Shown is one representative of 3 independent experiments. Bars indicate mean ± SEM. (**C**) A14 CTLs were co-cultured in ECM with HLA-matched primary CD4 targets (pulsed with 0 or 200 nM SL9 peptide). Antigen+/− samples were imaged in parallel at 1 min intervals for 10 hr with control samples imaged only at time 0 and 10 hr. Permeabilized target cells were assessed by CTXB^−^ sytox^+^ cell counts (left panel) and live CD8^+^ T cells were assessed by CTXB^+^ sytox^−^ cell counts (right panel). Shown is 1 representative of 4 independent experiments.

To directly observe the dynamics of CD8^+^ T cells engaging migratory target cells we developed a fluorescence videomicroscopy strategy for extended-duration imaging of cells within 3D type I collagen gels, simulating the 3D extracellular matrix environment of tissues. HIV-specific, HLA class I-restricted CD8^+^ T cells (primary cells and Gag-specific clones E501 and A14) were obtained from persons who spontaneously control HIV without the need for medication. These CD8^+^ T cells were co-cultured in collagen matrices with primary HLA-matched, activated CD4^+^ T-cells infected with HIV-1 or pulsed with cognate peptides. To distinguish target and effector cells, CD8^+^ T cells were labeled with fluorophore-conjugated B subunits of Cholera toxin (CTXB, the GM1-binding nontoxic subunit of CT, **Figure 1A**), which did not affect CD8^+^ T cell function in standard chromium release assays measuring CD8^+^ T cell killing of target cells (**Figure S1A in File S1**). We employed a high speed-motorized stage to support simultaneous imaging of up to 4 samples and acquisition of internally-controlled data sets.

Primary HIV-specific CD8^+^ T cell lines and the HIV Gag-specific CD8^+^ T cell clones A14 and E501 spontaneously polarized and migrated within collagen matrices with speeds and persistent random walk dynamics similar to those previously reported for T-cells migrating in 3D collagen *in vitro* [35] and tissues *in vivo* [3], [18], [19] (**Figure 1B** **in File S1 and Video S1 in File S2**). Sorted GFP^+^ CD4^+^ T cells infected with a GFP-tagged HIV construct (NL4-3-GFP) migrated in collagen with a reduced mean velocity relative to uninfected CD4^+^ cells, but ∼1/3 of the cells maintained speeds comparable to uninfected primary CD4^+^ T cells (6.0±0.37, mean ± SEM), similar to recent reports of HIV-infected human CD4^+^ T-cell migration *in vivo* in humanized mice [3]. (**Figure 1B**). When CTLs and uninfected CD4^+^ T cells were embedded together within collagen matrices using a ratio of one CD8^+^ effector to 2 CD4^+^ T cells (E:T 1∶2), antigen-independent CTL-target encounters were frequent (mean hit rate of ∼2 targets/hour) but brief (median of 7.2 min) and no killing of target cells or sustained adhesive interactions were observed (**Figure S1B,C** in File S1 and **Video S2 in File S2**). To visualize target killing, the DNA-binding dye sytox was added to the collagen matrix for *in situ* discrimination of live cells from dye-positive permeabilized cells (**Video S3 in File S2**); cell death was also typically obvious morphologically (**Video S4 in File S2**). When CTL clones were embedded in collagen matrices with Gag peptide-pulsed or control CD4^+^ target cells, videomicroscopy revealed antigen-dependent killing of targets by CD8^+^ T cells within collagen and continuous observation periods of at least 10 hrs were achieved with no evidence of phototoxicity to CTLs or killing of antigen-free CD4^+^ cells (**Figure 1C** and data not shown).

### Migrating HIV-infected Target Cells Frequently Escape from HIV-specific CTLs

**Figure 2:**
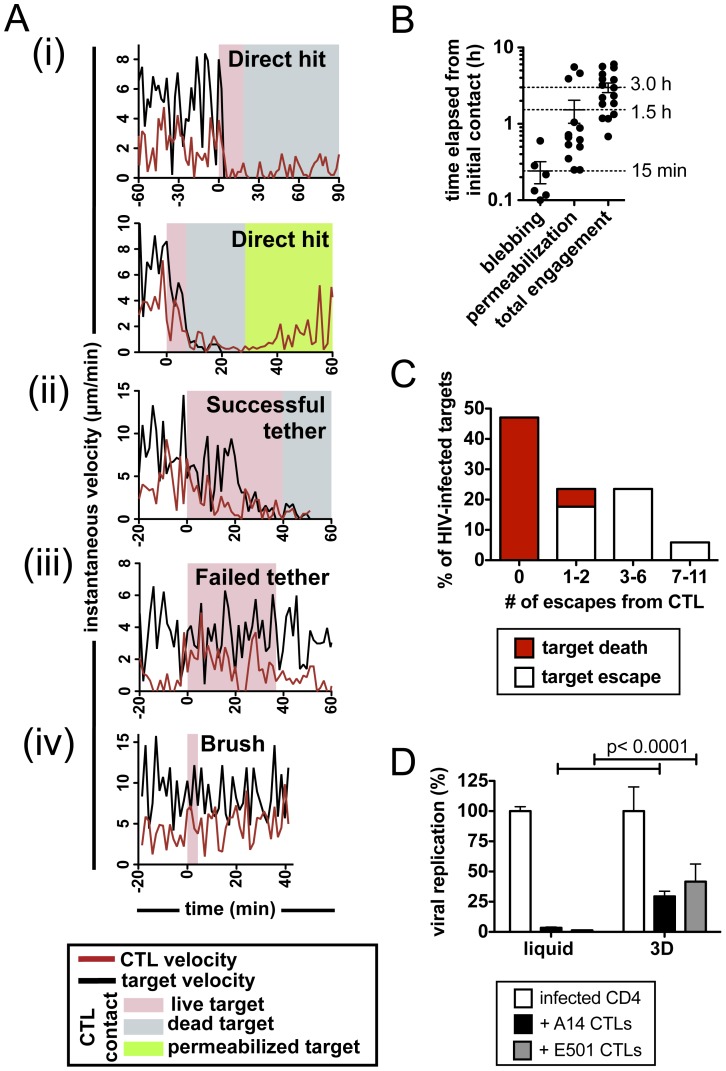
CTLs exhibit dynamic engagements with HIV-infected CD4^+^ target cells. (**A–C**) NL4-3-GFP HIV-infected CD4^+^ T cells (>95% GFP^+^) were imaged in collagen with E501 CTLs (E:T ratio 1∶2, 10 hr timelapse). (**A**) Contact types were characterized as (**i**) Direct hit kills, (**ii**) successful tethers, (**iii**) Failed tethers, and (**iv**) brushes. Shown are representative velocity traces (CTL in red, target in black) and the period of CTL-target engagement (live target in pink, morphologically dead target in gray, permeabilized target in green) vs. time for individual engagements of CTLs with infected targets. (**B**) Time elapsed from initial contact until blebbing and/or permeabilization and total duration of CTL-target contact is shown for engagements resulting in target death. (**C**) The number of escapes were enumerated for individual HIV-infected targets (n = 17 target cells analyzed). Engagements resulting in target death (red) or target escape (white) are indicated. (**D**) JR-CSF HIV-infected CD4^+^ T cells were cultured in liquid media or in ECM in the absence or presence of CTL clone A14 or E501 (E:T ratio 1∶1) and HIV replication relative to infected cells alone was assessed by p24 ELISA after 2 days. Data are from one representative of 3 independent experiments. Bars indicate mean ± SEM.

We first characterized the early dynamics of CTLs interacting with HIV-infected target cells, using the A14 and E501 CTL clones obtained from persons who spontaneously control HIV without the need for medications. HLA-matched CD4^+^ cells were infected with NL4-3-GFP, sorted by flow cytometry for GFP^+^ infected cells, then embedded with E501 CD8^+^ T cells in collagen matrices at a 1∶2 E:T ratio and imaged over several hours. Contacts between migrating CD8^+^ T cells and motile target cells led to 4 characteristic types of interaction (**Figure 2A**): During “direct hit” kills, CTL engagement with a motile GFP^+^ target triggered immediate motility arrest of both the effector and target cell, followed by rapid target cell death with or without permeabilization of the membrane (**Figure 2A** **(i), Video S5 in File S2**). In more rare instances, upon target engagement and CTL arrest, the target cell continued migration away from the arrested CTL, often pulling a long tail of target cytoplasm as it migrated. Eventually the tethered target stopped forward motion, blebbed, and snapped back to the CTL as it was killed, in these cases typically without permeabilization (“successful tethers,” **Figure 2A** **(ii), Video S6 in File S2**). However, not all CTL encounters with HIV-infected targets in the matrix led to target death: During “failed tethers” CD8^+^ cells adhered to a target and arrested, but the CD4^+^ cell continued to migrate, pulling long tails of target cell cytoplasm before escaping (**Figure 2A** **(iii), Video S7 in File S2**). Finally, “Brushes” were defined as microscopically observed contact between a motile HIV-infected target and a CTL, without CTL arrest or reorientation of the CTL toward the target (**Figure 2A** **(iv), Video S8 in File S3**).

Successful killing of infected targets was tightly linked with CTL motility arrest (**Figure S2 in File S1**) and killing progressed with a mean time of only 15 min to target blebbing and 92 min to permeabilization (**Figure 2B**). However, only ∼50% of E501 CTLs killed the first GFP^+^ target encountered in the matrix, despite the fact that >95% of rested CTLs were armed with stores of lytic granules (B27-KK10 tetramer^+^ GzmB^+^ by flow cytometry, data not shown). We thus examined the behavior of the infected targets more closely. While 47% of motile GFP^+^ targets were killed by the first CTL to engage them, other infected targets were engaged by multiple CTL over time but repeatedly escaped and continued to migrate during 10 hours of observation (mean of 4±3 escapes per unkilled GFP^+^ target, **Figure 2C**).

To relate the escape of migrating targets to ability of these CTLs to suppress virus in ECM, we next examined population-level CD8^+^ T cell function in an *in vitro* assay of viral replication [14]. The anti-viral activity of both E501 and A14 Gag-specific clones was compared within ECM gels and traditional liquid cultures, where targets and effector cells are in constant close contact and lack a migration-supporting matrix (**Video S1 in File S2**). Both clones strongly inhibited viral replication in liquid co-culture with infected CD4^+^ cells (**Figure 2D**). However, in parallel 3D collagen co-cultures, the CTLs were much less effective at limiting viral replication (p<0.0001, **Figure 2D**). Importantly, these results do not reflect a lack of CTL-target encounters within collagen as each CTL encounters an average of ∼2 target cells per hour in the matrix (**Figure S1C in File S1**). Thus, CTL antiviral efficacy is reduced within ECM, a finding that correlates with the ability of infected target cells to escape from migrating CTLs within a 3D matrix environment.

**Figure 3:**
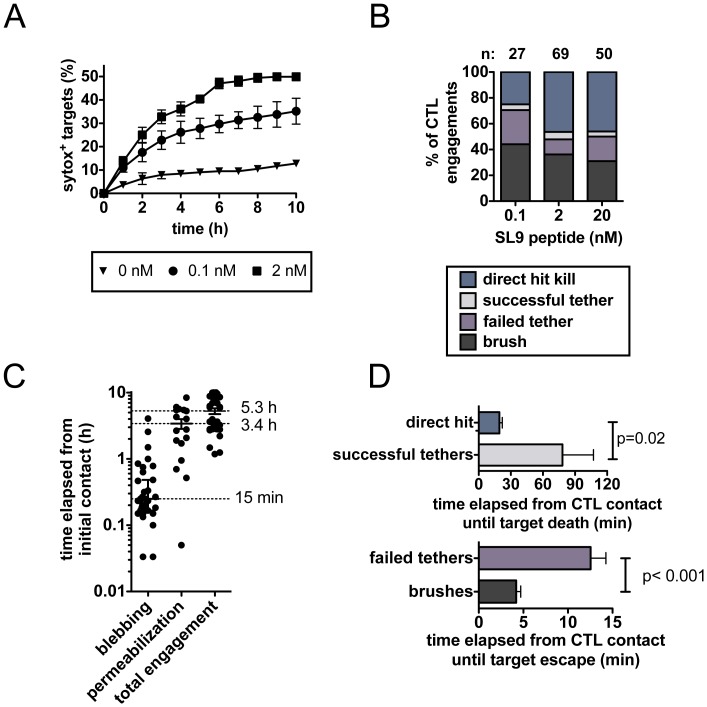
Peptide-pulsed targets elicit CD8^+^ T cell engagement dynamics similar to those observed for HIV-infected targets. A14 CD8^+^ T cells embedded with primary CD4^+^ T cell targets (pulsed with the indicated doses of peptide) within collagen gels were imaged for 10 hrs and analyzed for CTL-target engagement dynamics (E:T ratio 1∶2). (**A**) CTL killing activity (% sytox^+^ targets) vs. time as a function of antigen pulsing concentration. Background cell death in the absence of added antigen primarily reflected a low level of spontaneous target cell apoptosis early in the co-cultures. (**B**) Frequency of each type of CTL-target engagement during the first 30 minutes of co-culture as a function of antigen pulse concentration. (**C**) Time elapsed from initial contact until blebbing and/or permeabilization and total duration of CTL-target contact is shown for engagements resulting in target death (targets pulsed with 20 nM SL9 peptide). (**D**) Durations of characteristic CTL-target engagements determined for targets pulsed with 20 nM SL9 peptide.

To confirm that the dynamics observed with HIV-infected cells were independent of infection-mediated changes in target cell physiology, we carried out similar imaging analysis utilizing CD4^+^ targets pulsed with low nanomolar concentrations of synthetic HIV peptides. The frequency of killing contacts for A14 CTLs encountering targets (pulsed with cognate peptide Gag-SL9), as evidenced by uptake of sytox, was antigen dose dependent, (**Figure 3A–B**), elicited the same killing/escape contact dynamics (**Figure 3B**), and the kinetics of killing events were similar to those observed in response to HIV-infected targets (**Figure 3C**). Further analysis of the duration of CTL-target engagements revealed quantitatively different periods of contact for each dynamic/outcome: direct hits on peptide-pulsed targets progressed to target death in ∼15–20 min, while killing of tethered targets was much slower, taking on average 80 min (**Figure 3D**). By contrast, antigen-pulsed targets that escaped from CTLs were in contact for only ∼12 min in failed tethers or <5 min in brushes (**Figure 3D**). Qualitatively similar results were obtained for the E501 CTL clone (which recognized the synthetic gag KK10 peptide, data not shown) and polyclonal primary CD8^+^ T cell lines (which recognized pools of overlapping 18-mer peptides derived from the sequences of gag, env, nef, and pol, **Video S9 and S10 in File S3**). Taken together these data indicate that these dynamics are CTL-intrinsic and apply not only to CTL clones but also freshly isolated CD8^+^ T cells from infected persons, and do not reflect HIV-specific alterations in target cells.

### Target Cell Motility Impedes Antigen Recognition and Directly Supports Target Escape

**Figure 4:**
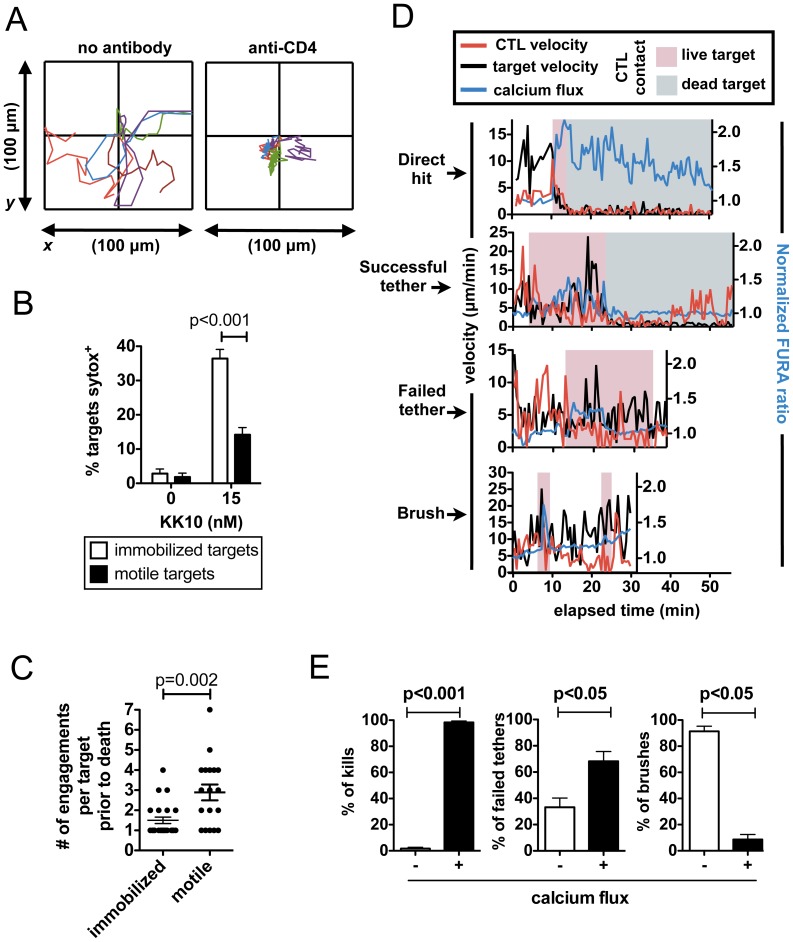
Target cell motility directly impacts CTL function. (**A–C**) CD4^+^ target cells (controls or pulsed with KK10 gag peptide) were spun to the bottom of collagen matrices prior to gelation to allow binding to the underlying glass substrate, which was coated with anti-CD4 antibody and ICAM or ICAM alone. E501 CTLs were added to the matrix (E:T ratio 1∶2) and target/CTL dynamics were recorded by videomicroscopy for 10 hr. (**A**) Wind-rose plots of target cell migration over a period of 20 min in the absence or presence of immobilizing anti-CD4. (**B**) Target cell death was assessed by sytox fluorescence after 10 hr. (**C**) Engagement histories were recorded for motile or immobilized targets that were killed. (**D, E**) A14 CTLs were loaded with FURA-2 AM and Ca^2+^ signaling was monitored for CTLs engaging peptide-pulsed CD4^+^ targets (2 nM SL9, 1 hr time-lapse). (**D**) Shown are representative Ca^2+^ traces (blue) and instantaneous cell velocities (CTL in red, target in black) over time on the x-axis. The period of CTL-target engagement is denoted on the time axis (live target in pink, killed in gray). (**E**) The presence or absence of Ca^2+^ signals was scored for CTL-target engagements concluding with target death (*n* = 89), a failed tether (*n* = 112), or a brush (*n* = 95). Data pooled from 6 independent experiments. Bars indicate mean ± SEM.

To determine whether the motility of target cells within the 3D matrix environment *per se* reduced CD8^+^ T cell efficacy, peptide-pulsed target cells were either allowed to migrate freely or were immobilized on the glass substrate at the base of collagen gels with anti-CD4 antibodies (**Figure 4A**). E501 CTL clones lysed 2.6-fold more immobilized target cells than freely motile target cells after 10 hr (p<0.001, **Figure 4B**). Analysis of the number of CTL engagements prior to target death revealed that immobilized targets were most often killed upon their first encounter with a CTL, but motile CD4^+^ targets escaped a mean of 2.9±0.4 CTLs before being killed (**Figure 4C**). Death of non-immobilized CD4^+^ cells progressed with similar kinetics whether targets received a lethal hit from the first CTL encountered or the *n*th CTL (following escape from multiple CTLs), suggesting that failed CTL-target engagements did not inflict cumulative, sub-lethal damage (**Figure S3A in File S1**). The significant impact of target cell motility on the efficiency of CTL killing was also observed for EBV-transformed B cells bearing the same HIV peptide epitopes (**Figure S3B in File S1**). Together these data indicate that target migration directly impacts the efficiency of killing by CTLs in 3D matrix.

CTL triggering and synapse formation is mediated by an orchestrated rapid series of receptor engagements and downstream signaling cascades. To determine where in this process CTL triggering fails during target cell escape, we next characterized the impact of target cell motility on intracellular calcium signaling, one of the earliest events downstream of TCR triggering. A14 CTLs labeled with Fura-2AM engaging peptide-pulsed CD4^+^ targets elevated intracellular calcium coincident with CD8^+^ T cell migration arrest during both direct hit kills and successful tethers, but direct hits were accompanied by calcium fluxes of greater amplitude and duration that persisted post-target death (**Figure 4D, E** and **Video S11 in File S3**). In contrast, a weak calcium flux was triggered in only 30% of CD8^+^ T cells engaging in failed tethers, which terminated prior to target cell escape (**Figure 4D, E** **and Video S12 in File S3**). Signaling was largely absent during brushes (**Figure 4D, E**). Thus, tethering contacts with migrating targets lasting tens of minutes did not ensure full engagement of downstream TCR signaling, and target cell escape was accompanied by a failure of strong/sustained calcium signaling to be triggered in the CTL.

### High TCR Avidity is Required for Efficient Engagement and Killing of Targets in ECM

**Figure 5:**
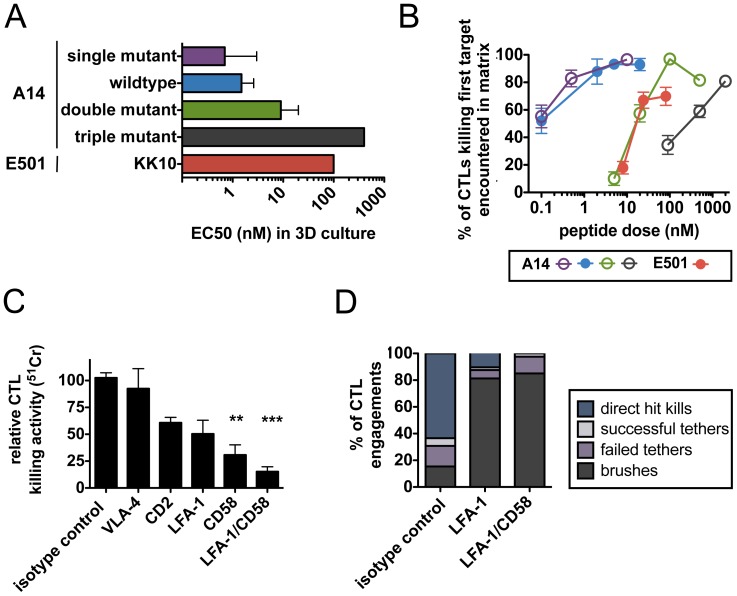
High antigen sensitivity and adhesion receptors CD58 and LFA-1 are required for efficient killing of motile targets by individual CD8^+^ T cells. CD8^+^ T cell clones were co-cultured in ECM with HLA-matched CD4^+^ targets (E:T ratio 1∶2). (**A**) Functional avidities of A14 and E501 CTLs engaging targets in collagen were determined by ^51^Cr release assays, by determining EC_50_s (antigen pulsing concentration required for 50% of max target death, for SL9 peptide and SL9 variants or KK10 peptide, respectively). (**B**) CTL efficiency was observed at the single-cell level (% of individual CTL successfully engaging the first motile target encountered). Data pooled from 3–5 independent experiments, *n* for each condition within each experiment was 10–30 CTL. Bars indicate mean ± SEM. (C–D) A14 CTLs were co-cultured with HLA-matched CD4^+^ targets (pulsed with 2 nM SL9) within ECM in the presence of the indicated adhesion receptor antagonists (10 µg/mL neutralizing antibody or 50 nM inhibitor) or isotype and vehicle controls. (**C**) CTL killing activity was measured by ^51^Cr release from targets after 6 hr of co-culture within ECM. Data pooled from 3 independent experiments. Bars indicate mean ± SEM. (**D**) Frequencies of characteristic A14 CTL-target engagements during the first 30 minutes of videomicroscopy.

Our data suggest that within extracellular matrix, active migration of CD4^+^ targets away from CTLs reduces the efficiency of target cell killing, yet CD8^+^ T-cell responses can suppress viral replication *in vivo* [9], [36], [37]. Since high functional avidity of CTLs correlates with efficient viral suppression *in vitro* [38], we tested the impact of CTL avidity at the single-cell level by measuring the efficiency with which CTLs killed the first target cell contacted in the matrix. To vary avidity in the absence of inter-clonal differences in CTLs, we employed a panel of SL9 peptide variants known to develop in HIV-infected patients that bind equivalently to HLA-A2 [39]. In bulk chromium lysis assays in collagen, the A2-restricted A14 CTLs recognized HLA-matched CD4^+^ target cells pulsed with these SL9 variants with EC_50_s varying from 0.7 nM to 400 nM (**Figure 5A**). Strikingly, videomicroscopy of these co-cultures revealed that A14 CTLs achieved first-contact kill efficiencies of >90% in response to targets pulsed with low concentrations (1–10 nM) of the SL9 peptides recognized with high avidity (single mutant or wild-type). However, target cells bearing peptides recognized with lower avidity showed a much higher frequency of escape from these same CTLs, requiring ∼10- and ∼1000-fold higher doses of double mutant or triple mutant peptides, respectively, to elicit comparable first-contact killing efficiencies (**Figure 5B**). Similarly, E501 CTLs, restricted by HLA-B*2705 and exhibiting ∼65-fold lower avidity for their cognate peptide than A14 CTLs (**Figure 5A**), required nearly 100-fold higher antigen doses to achieve >50% first-contact-kill efficiency against migrating targets in collagen (**Figure 5B**). These data indicate that even when antigen is limiting, high avidity CTLs are capable of catching and killing the first target encountered in a 3D matrix. Conversely, low avidity CTLs routinely failed to successfully engage migrating targets at low antigen densities.

### Adhesion Receptors CD58 and LFA-1 are Critical for CTLs to Catch and Kill Targets in ECM

The presence of target cell tethering suggested that effective target cell adhesion is a critical component of CTLs engaging targets in a 3D tissue matrix. We thus quantified the importance of key CTL/target adhesion molecules in mediating motile target cell capture. Both A14 CTLs and primary CD4^+^ T-cell targets expressed CD2, CD58, LFA-1, and ICAM-1 (data not shown). Addition of blocking antibodies (for CD2, CD58, ICAM-1) or small-molecule antagonists (for LFA-1 and the collagen receptor VLA-4) to collagen matrix bulk killing assays showed that VLA-4 was not important for A14 CTL recognition of SL9-pulsed target cells, but each of the cell-cell adhesion receptors tested played a role in target cell killing, with combined blockade of LFA-1 and CD58 leading to a ∼85% reduction in killing (**Figure 5C**). Videomicroscopy analysis of CD8^+^ T cell engagements with CD4^+^ targets in the presence of adhesion receptor blockade showed that the frequency of A14 mediating direct hit kills decreased by ∼5-fold and the frequency of first-contact-kills decreased by ∼4-fold when LFA-1 was blocked compared to vehicle-treated controls; combined LFA-1/CD58 blockade nearly eliminated all target cell capture/killing (**Figure 5D**). Thus, CD8^+^ T cells exhibiting high functional avidity can effectively capture migratory target cells in extracellular matrix, in a manner dependent on effective adhesion receptor engagement.

### CTL Remain Committed to Killed Targets for Many Hours

**Figure 6:**
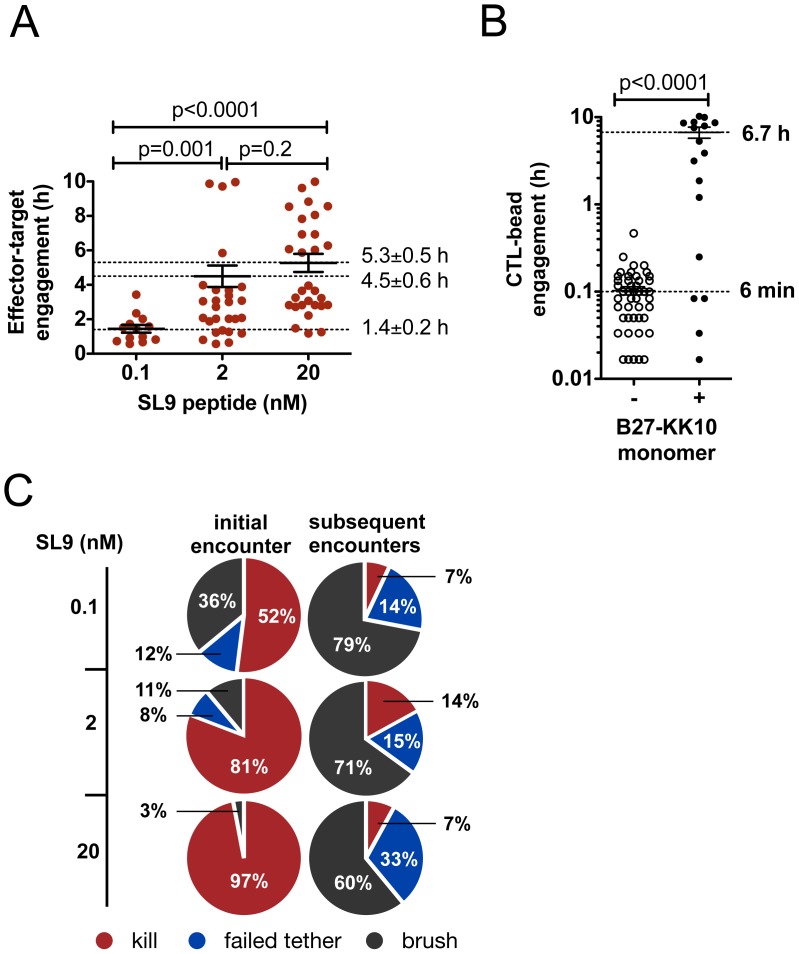
CTL exhibit a prolonged TCR-dependent, CTL-intrinsic migration arrest after successfully engaging and killing an initial target. (**A**) A14 CTLs were co-cultured in collagen with CD4^+^ target cells pulsed with SL9 peptide (E:T ratio 1∶2) and total engagement times for CTL-target encounters ending in target death were recorded. Data shown from 1 representative of 5 independent experiments. Bars indicate mean ± SEM. (**B**) The duration of E501 CTL engagements with 15 µm beads presenting recombinant B27-KK10 peptide-MHC complexes at a density of 20 pMHC/µm^2^ or control beads lacking pMHC in collagen was quantified by videomicroscopy. Data are from 1 representative of 4 independent experiments. Bars indicate mean ± SEM. (**C**) A14 CTLs were imaged in collagen with CD4^+^ T-cells pulsed with the indicated doses of SL9 peptide in collagen (E:T ratio 1∶2, 10 hr timelapse). Outcomes for each CTL engaging its first target or subsequent targets are expressed as % of first CTL encounters or % of subsequent encounters (*n* = 185 (0.1 nM SL9), 147 (2 nM SL9), and 308 (20 nM SL9) engagements analyzed). Data are pooled from 3 independent experiments.

Using the ability of the videomicroscopy assay to permit time-lapse imaging of CTL-target interactions in collagen over durations of many hours, we next characterized events occurring beyond the first few encounters of CTLs with targets. Strikingly, CTLs that engaged and killed HIV-infected targets remained arrested in prolonged contact with dead targets for much longer (mean of 3.0±0.4 hrs) than the time required for delivery of a lethal hit (14.5±4.6 min to blebbing) (**Figure 2B**). Using Gag peptide-pulsed target cells, the duration of engagement with killed targets was found to be antigen dose-dependent, and the duration of CTL-dead target contacts increased with increasing antigen density (**Figure 6A**). At antigen doses eliciting substantial killing, more than 10 hrs was required for CTL migration to resume (**Figure S4A in File S1**), and disengaged CTL often remained in the vicinity of their killed target for many hours (**Video S13 in File S3**). Similar dynamics were observed for primary HIV-specific CTL encountering CD4^+^ T cell targets and for the lower-avidity E501 clone encountering CD4^+^ T cell targets and BCL targets (data not shown). To determine whether TCR engagement alone was sufficient to induce this long-lived arrest, we displayed recombinant B27-KK10 peptide-MHC complexes on cell-sized microspheres and analyzed the contact times of CD8^+^ T cells with these beads in collagen. As shown in **Figure 6B**, E501 CTLs migrating into contact with pMHC-displaying beads arrested and remained engaged with beads for 6.7±1 hours, but interacted with control beads for only 6±0.7 min indicating that prolonged arrest was a CD8^+^ T cell-intrinsic response to TCR engagement.

Following commitment to an initial killed target, CD8^+^ T cells engaged subsequent targets inefficiently. This was not due to a lack of encounters or insufficient periods of observation since targets frequently migrated over these arrested CD8^+^ T cells. Occasionally, arrested CTLs caught and killed an additional CD4^+^ cell within minutes or hours of killing their initial target (**Figure 6C** **, Figure S4B in File S1 and Video S14 in File S3**), but the large majority of encounters occurring over >10 hr between migrating targets and arrested CD8^+^ T cells post-first-kill were failures (brushes or failed tethers) (**Figure 6C**). Flow cytometry analysis of CTLs co-cultured with targets indicated that, at the antigen doses used, CTLs had not depleted their stores of lytic granules (**Figure S4C in File S1**). Thus, after efficiently killing a target, CD8^+^ T cells exhibit a TCR-dependent, CTL-intrinsic program marked by many hours of arrested motility and inefficient engagement of additional targets.

### CD8^+^ T Cells Transition to Sustained Non-lytic Effector Secretion during Prolonged Arrest

**Figure 7:**
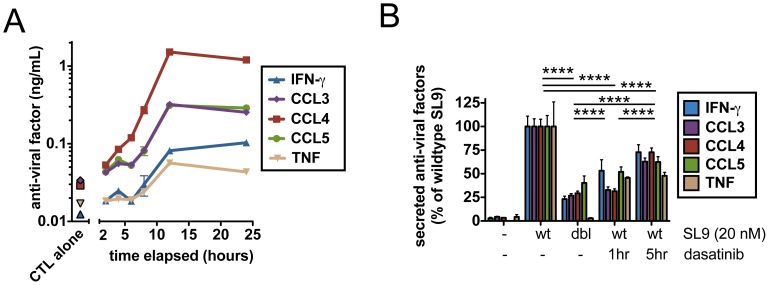
CTLs rapidly transition from killing to sustained non-lytic effector secretion during prolonged arrest. A14 CTLs and peptide-pulsed CD4^+^ target cells (20 nM SL9) were co-cultured in collagen (E:T ratio 1∶2) and supernatants were harvested after the indicated time periods. Concentrations of secreted cytokines/chemokines were analyzed using Flex Set bead-based ELISAs (BD Biosciences). Each data point reflects a unique set of triplicate wells for each timepoint or condition. (**A**) Kinetics of cytokine/chemokine secretion were determined for CTL-target co-cultures or for CTLs incubated alone over a 24 hour time period. (**B**) Target cells were pulsed with SL9 or double mutant SL9 peptide and co-cultured in the presence or absence of the TCR signaling inhibitor dasatinib added after 1 hr or 5 hr of co-culture; secreted cytokine concentrations were assessed as in (A). **** p<0.0001. Significance of data was determined by a 2 way ANOVA followed by a Bonferroni post test. Data shown are from 1 representative of 3 independent experiments. Bars indicate mean ± SEM.

As CTL clones cultured in collagen with NL4-3-infected CD4^+^ cells produced substantial quantities of inflammatory chemokines and cytokines by 12 hrs (data not shown), we hypothesized that prolonged arrest on dead targets might reflect a transition of engaged CTLs from a lytic to secretory phase. As noted above, CD8^+^ T cells arrested on dead targets showed continued calcium signaling after target death (**Figure 4D**). To determine whether initial killing/CTL arrest coincided temporally with induction of effector secretion, we measured the kinetics of cytokine/chemokine secretion by CD8^+^ T cells engaging peptide-pulsed target cells in collagen. At an antigen dose (20 nM SL9) where >95% of A14 clones engaged a target and arrested within 30 min of co-culture (**Figure S5 in File S1**), cytokine and chemokine secretion was detected after 2 hr, as previously reported for CTL in liquid cultures [12]. Secretion continued for at least 12 hr while these CD8^+^ T cells remained arrested, leading to steady accumulation of cytokines/chemokines in the culture (**Figure 7A**). However, if TCR signals were attenuated by addition of an inhibitor of TCR signaling (dasatinib) after 1 or 5 hours of co-culture, or by co-culture of CTLs with targets bearing low-avidity peptides (20 nM double mutant SL9), then CD8^+^ production of anti-viral cytokines and chemokines was reduced (**Figure 7B**). Taken together, these data indicate that CD8^+^ T cells require strong, prolonged TCR signals for maximum effector secretion. Furthermore, they coordinate their lytic and non-lytic effector functions spatiotemporally in ECM; target cell killing is followed by sustained arrest on dead targets and upregulation of anti-viral effector molecule secretion.

## Discussion

Here we recorded the behavior of individual HIV-specific CD8^+^ T cells starting with their first antigen encounter and continuing for prolonged periods, up to 10 hr, within a 3D model of peripheral tissue. This approach supported analysis of both short- and long-term cellular dynamics, the latter of which are presently inaccessible *in vivo*. This analysis revealed a coordinated program of CD8^+^ T cell functions and migration dynamics that evolves over time: Migrating CD8^+^ T cells surveyed 3D ECM in search of antigen and, upon successful recognition, exhibited two phases of antiviral function: a rapid “commitment phase” (target engagement, adhesion, TCR signaling, migration arrest, target killing) followed by a prolonged “secretory phase” (prolonged migration arrest, prolonged TCR signal accumulation, sustained effector molecule production).

While CD8^+^ T cell killing and secretory functions have been well studied *in vitro*, how these functions are integrated with cell migration dynamics has remained unclear. We found that HIV-specific CD8^+^ T cells in ECM exhibit the initial cascade of prototypical TCR-mediated events [23], [40] and killing kinetics [24], [28], [29] previously described for CTL in liquid cultures *in vitro*. However, we also observed that migrating target cells are capable of escaping from CTLs in ECM. Further, CD8^+^ T cells making a successful kill did not immediately detach from dead targets upon lethal hit delivery within ECM. Instead, CD8^+^ T cells remained arrested (frequently >10 hr) and largely failed to engage and kill subsequent passing targets, expanding upon short-term (30–60 min) *in vivo* studies that report CTL arrest associated with killing [18], [21], [41], [42], [43]. It should be noted that in studies of CTL function, high antigen densities (achieved by pulsing targets with micromolar concentrations of peptide) are frequently employed to guarantee a measurable response. However, in the setting of HIV, endogenously processed viral peptides can be presented at very low antigen densities on target cells [44], [45]. Thus, we focused on the dynamics and function of the CTL response to HIV-infected targets presenting physiologically relevant antigen and targets bearing limiting doses of antigen (low nanomolar concentrations of peptide).

HIV-infected CD4^+^ T cell migration has dire consequences *in vivo*, supporting HIV replication and dissemination of viral infection [2]. Here we have shown that motility directly supports target escape even when CD8^+^ T cell contact occurs. However, CD8^+^ T cells can and do contribute to spontaneous control of HIV in rare individuals [7], [32], [46], [47], [48] and vaccine-induced control in non-human primates [7], [8], [9], [49]. Multiple dimensions of CD8^+^ T cell activity have been correlated with control of HIV *in vivo*: these include high lytic capacity [48], functional avidity [50], β-chemokine expression [51], [52], polyfunctionality (i.e. lytic degranulation and non-lytic anti-viral effector molecule production) [8], [46], TCR usage [47] and proliferative capacity [48], [50].

Although these correlates of effective CD8^+^ T cell function have been made, the current view of effector functions is kaleidoscopic. An integrated program describing how individual CD8^+^ T cells coordinate their multiple functions to eliminate a viral infection remains unclear. We set out to define parameters that regulate CD8^+^ T cell functions within 3D ECM over a 10 hr time-scale encompassing early lytic and late secretory functions. Analysis of these dynamics at the single-cell level revealed a new role for TCR avidity in regulating the function of individual CD8^+^ T cells in the context of cell migration: High-avidity cells were capable of near-perfect efficacy in capturing and killing migrating targets on first contact, while target cell motility facilitated escape from CD8^+^ T cells under conditions of weak antigen recognition (low antigen dose or low-avidity TCR/peptide-MHC interactions). Interestingly, adhesion molecules LFA-1, CD2 and CD58 mediate antigen-independent CTL-target conjugate formation in liquid cultures [53] and LFA-1 is required for efficient TCR-dependent killing mediated by directed release of lytic granules towards the target cell [54]. Here we have shown that these molecules are required to not only to support efficient antigen-dependent CTL killing but also to prevent the escape of motile targets in a 3D environment.

Furthermore, we found that the duration of effector-target engagement is not pre-programmed but rather is controlled by TCR signal strength, and that TCR signal accumulation continues post-target-death. In liquid cultures, net TCR signals are integrated over time [55] and CD8^+^ T cell functions are induced according to a hierarchy of increasing TCR signaling thresholds [28], [32], [56]. Our data suggest that strong early TCR signals associated with killing trigger a positive feedback loop of prolonged arrest, permitting greater net TCR signal accumulation that in turn promotes greater induction and sustained secretion of effector cytokines and chemokines (at least 12 hr). By contrast, weak TCR signaling both makes initial target killing less efficient and also results in poor effector function since TCR signal accumulation is prematurely cut short by abandonment of the dead target. Thus, strong TCR stimulation drives a program of spatially coordinated, multidimensional (i.e., combined lytic and secretory) CD8^+^ T cell function. Note that although our analysis shows that T cell functional avidity is important, other aspects remain important, such as the antigen targeted by the CTL: For example, although the A14 CTL showed very efficient killing of peptide-pulsed targets at very low antigen densities, it remained only modestly better than the low-avidity E501 CTL clone in controlling viral replication in collagen. This result is consistent with differences in the antigen processing and presentation of the two epitopes in infected cells [45].

The 3D ECM model system permitted detailed analysis of the TCR-dependent CD8^+^ T cell-intrinsic program of functions, however these results must be interpreted within the limitations of our reductionist approach. We established conditions where the phenotypic and functional characteristics of targets and killer cells were well defined. Only two virus-specific clones and freshly-primed CD8^+^ T cells from one patient were examined, but all gave concordant results. This model system eliminates many variables present *in vivo*, and many additional immune cells and mechanisms contribute to the spread and/or control of virus *in vivo* [57]. For example, the very long commitment of arrested CTLs to dead targets observed here may be truncated at some time in vivo by macrophages clearing apoptotic target cells. Since it is clear that CD8^+^ T cells have the capacity to kill multiple targets if targets are encountered simultaneously [31] and commit rapid, “serial kills” in suspension culture [24], [58], [59], [60], we revisited this potential mechanism in the present model. Individual CD8^+^ T cells were observed for at least 10 hours, but killing of serially encountered targets was rarely observed. Other cells or soluble factors could be present in infected tissue that could influence cell motility and/or CTL function, but notably, addition of potent motility-driving chemokines such as CCL21 did not alter the sustained stop signal behavior of CD8^+^ T cells here (data not shown).

Together, our findings lead us to propose a two-phase model for control of infection by individual virus-specific CD8^+^ T cells exhibiting cooperative behavior on the population level: On detecting antigen, rather than attempting to control infection individually through serial lytic hits, high-avidity CD8^+^ T cells efficiently engage and kill initial targets (commitment phase). Killing is followed by a rapid transition to non-lytic effector function, bathing the local microenvironment with antiviral inflammatory factors (secretory phase). In this scenario, sustained secretion of β-chemokines may rapidly shift the local E:T ratio, by blocking infection of new targets and recruiting additional “ready-to-kill” effectors into the tissue. Conversely, a population of low-avidity CD8^+^ T cells will exhibit multifaceted failure: motile targets will escape inefficient engagement, permitting viral replication and spread, while upon killing a target, weak TCR signaling and secretion of low quantities of β-chemokines insufficient for blocking new infections might recruit additional CD4^+^ cells to the tissue, thereby inadvertently promoting rapid viral replication [2]. This mechanism for spatiotemporal coordination of antiviral effector functions is dependent on CD8^+^ T cell killing efficiency and highlights a new role for TCR avidity in tissue where effector cells and target cells are motile. Thus, a successful HIV vaccine employing CD8^+^ T cells would need to induce high-avidity CTL, which can mount a cooperative and multidimensional response for efficient elimination of migrating infected cells prior to overwhelming virus dissemination.

## Materials and Methods

### Ethics Statement

The institutional review boards of Massachusetts General Hospital and Massachusetts Institute of Technology approved the studies of cells derived from human blood samples and all human subjects gave written, informed consent.

### Human Subjects

PBMCs were obtained from healthy donors (Research Blood Components) and HIV-infected persons through outpatient clinics at Massachusetts General Hospital, Brigham and Women’s Hospital, and Fenway Health in Boston, MA. Primary HIV-specific CTL were derived from subject 285873, an elite controller with known HLA (A0201/A0301, B5101/B5101, Cw1402/Cw1502), low viral load (<75 virions/mL), strong A2-SL9 specific CTL responses (3600 SFC/10^6^ PBMCs), and a broad CTL response to optimal peptides (A2-SL9, A3-KK9, A3-RK9, A3-RY10, Cw14-LL8 (p17), A2-VV9 (p24), A3-TK10 (gp120), A3-RR11 (gp41), A2-SAV10 (env), A3-QK10 (nef), B51-LI9 (int), B51-EI9, (vpr), B51-LI9 (gp160)) and individual 18-mer peptides (recognizing 26/48 Gag, 9/13 Nef, 5/6 Tat, 8/24 Pol, 0/4 Vpr, 1/14 Env, 0/3 Vif overlapping 18-mer peptides) in traditional ELISPOT assays. The E501 clone was derived from subject 828656, an elite controller with known HLA (A0301/2601; B1501/2705; Cw0202/0303), pVL <50 copies/ml, CD4 count 717 cells/µl, dominant B2705-KK10 responses with 3.68% KK10 tetramer^+^ cells in CD3^+^CD8^+^ population. A2-SL9 clone A14 was generated from a controller with known HLA (A0201/A0301, B0702/B4001, Cw0304/Cw0702), low viral load (96 copies/mL), strong A2-SL9 specific CTL response (750 SFC/106 PBMCs), and a broad CTL response to optimal peptides (A2-SL9 (p17), A2-KK9 (p17), A3-RK9 (p17), A3-RR11 (gp41), B7-IL9 (gp41), B7-GL9 (p24), B7-RV9 (nef), B40-IL10 (p17), B40-SL9 (p24), B40-IL9 (RT), B40-QL10 (gp41), B40-KL9 (nef), Cw7-RY11 (nef) in ELISPOT assays.

### HIV-1 Virus Production

#### VSV-g pseudotyped virions

HEK293T cells were transfected with pHDM-G vsv-g (Dr. Jeng-Shin Lee, Harvard Gene Therapy Initiative) and pBR-NL43-IRES-eGFP-nef^+^ [61] or pNL4–3 (AIDS Research and Reference Reagent Program, NIAID, NIH; Cat #11349 deposited by Dr. F. Kirchhoff, or Cat #114 deposited by Dr. M. Martin, respectively) with LipoFectamine 2000 (Invitrogen) according to manufacturers’ instructions. Viral supernatant was harvested after 48 hr and stored in aliquots at −80°C.

#### JR-CSF

Activated CD4^+^ T cells were infected with JR-CSF (MOI 0.001) and incubated for 4–6 days. Viral supernatants were stored in aliquots at −80°C.

### Preparation of HIV-specific CTLs and Target Cells

#### CTL clones

The A14 (also referred to as 161jxA14) clone and the E501 clone were previously described [62], [63] and are specific for A02-SL9 (gag p17) and B27-KK10 (gag p24), respectively. CTL clones were maintained with periodic restimulation and used in assays 12–20 days post-restimulation. For imaging, CTLs were stained with 5 µg/mL Alexa 647-CTXB (Invitrogen) in R10/50 medium for 30 min at 37°C, 5% CO_2_, and washed twice. For calcium imaging, CTL were simultaneously labeled with CTXB and Fura-2 AM (Invitrogen) at 20 µg/mL for 20 min at 37°C, 5% CO_2_ and imaged according to the manufacturer’s instructions.

#### Primary polyclonal HIV-specific CD8^+^ T cells

CD8^+^ T cells from individual 285873 were negatively selected (EasySep, StemCell Tech.) from thawed PBMCs and primed with HLA-matched CD4^+^ T cell targets pulsed with pools of overlapping 18-mer peptides spanning the HIV-1 proteins Gag, Pol, Nef, Env. Cultures received IL-2 (50 U/mL) on days 2, 5 and 12. Polyclonal CD8^+^ T cells were used in assays on day 12.

#### Primary CD4^+^ T cell targets

CD8^+^ cells were separated from PBMCs by positive selection (EasySep, StemCell Tech.) and non-CD8 cells were activated with anti-CD3∶8 (JT Wong, Harvard Med. School), anti-CD28 (R&D Systems), and IL-2 for 5–7 days to generate primary CD4^+^ cell targets. Where indicated, CD4^+^ T cells were pulsed with peptide for 1 hr at 37°C and washed. Alternatively, CD4^+^ T cell targets were spinfected in R10/50, polybrene (8 µg/mL) and HIV-1 (MOI of 0.001 to 0.1) at 37°C, 2400 RPM (1220 g) for 2 hr and washed. For imaging studies, infected populations were used directly when ∼30% of the cells were p24^+^ by flow cytometry (∼3 days post-infection), or as a purified infected population (>95% NL4-3-GFP^+^) 3 days post-infection.

#### EBV-transformed B cell targets

Where indicated, BCL expressing HLA-B27 were pulsed with KK10 (KRWIILGLNK, for which 1 nM peptide is equivalent to 1.25 ng/mL) for 1 hr at 37°C, washed and used immediately in killing assays.

### Peptides

SL9 (SLYNTVATL, or variants) for which 1 nM peptide is equivalent to 1 ng/mL, and KK10 (KRWIILGLNK) for which 1 nM peptide is equivalent to 1.25 ng/mL were used at the indicated concentrations. Overlapping 18-mer peptide pools spanning the HIV-1 proteins Gag, Pol, Nef, Env were used at concentrations of 100 ng/mL.

### Viral Inhibition Assays

Primary activated CD4^+^ T cells spinfected with the R5-tropic JR-CSF (MOI 0.01) were used as targets for their susceptibility to both lytic and non-lytic (β-chemokine mediated inhibition of new target infection via CCR5) CD8^+^ T cell functions. Targets were established co-culture with CD8^+^ T cell clones immediately following spinfection (E:T ratio 1∶1). After 0 and 2 days of culture in liquid or ECM in flat 96-well plates, triplicate samples were lysed in wells with 1% triton X-100. HIV concentrations were determined by p24 ELISA (Perkin Elmer) and viral replication was calculated. The p24 concentrations from infected CD4^+^ T cells alone measured on day 0 and day 2 were used to define 0% and 100% replication, respectively.

### Chromium Release Cytolysis Assays

CD4^+^ T-cell targets were ^51^Cr labeled (250 µCi/mL) and peptide-pulsed for 1 hr at 37°C. Liquid and 3D collagen assays were performed in triplicate in round-bottom 96 well plates at an E:T ratio of 1∶1 (1×10^4^ cells each) in a total volume of 200 µl. Co-cultures and controls (MaxR, targets lysed with 3% Triton-X 100; SR, targets alone) were packed by centrifugation (3 min, 300 g) and incubated at 37°C, 5% CO_2_ for 4–6 hours. ^51^Cr was measured in supernatants on a Packard Topcount. EC_50_s were calculated following nonlinear regression (Prism, Graphpad Software). % killed = (CPM−mean SR)/(mean MaxR CPM−mean SR CPM)*100.

### Time-lapse Microscopy of T-cells in 3D Collagen Matrix

8-well chambered coverglasses (Labtek, Nunc) were coated with 100 µg/ml fibronectin in PBS for 18 hr at 4°C, washed, and pre-warmed (37°C) just prior to use. Neutral solutions of type I bovine collagen (PurCol 5005, Advanced Biomatrix) containing sytox green (Invitrogen, 5 µM in the final gel), RPMI, 10% FCS were prepared following the manufacturer’s instructions on ice. Cells (4×10^4^ effectors, 8×10^4^ targets) suspended in 400 µL neutral collagen were deposited in coverglasses, centrifuged at 300× *g*, 3 min. Samples were immediately imaged on a Zeiss Axiovert 200 epifluorescence microscope equipped with environmental control (37°C, 5% CO_2_). Three images (sytox, CTXB, brightfield) were collected every 1 min at 20X or 40X for 4 adjacent stage positions in 4 parallel samples per imaging run (total field of view 1340×1800 µm per sample).

### Immobilized Target Studies

8-well coverglasses (Labtech #1 glass, Nunc) were coated with anti-CD4 (20 µg/mL OKT4 and RPA-T4, eBioscience) with or without rhICAM-1 (1 µg/mL, R&D Systems) in PBS at 4°C for 18 hr. Slides were washed and prewarmed (37°C) prior to use. Cells in collagen were then added to coated coverglasses as described above.

### Peptide-MHC-I Displayed on Cell-sized Beads

Streptavidin beads (Bangs Labs, CP01N, 15 µm) were rotated at 4°C in the presence of biotinylated monomeric peptide-MHCI complexes (KRWIILGLNK, HLA-B*2705: Dale Long, NIH Tetramer Core Facility, Emory University) in PBS. After 2 hours, the beads were washed with PBS, 0.1% BSA and used immediately. The density of peptide-MHC-I on the surface of beads (20 pMHC/µm^2^) was quantitated by staining with PE-HLA-ABC (BD Biosciences) and comparing to Quantum Simply Cellular anti-Mouse IgG beads (Bangs Labs, 815) according to the manufacturer’s instructions.

### Videomicroscopy Data Analysis

Image analysis was carried out using Metamorph (Molecular Devices) and ImageJ (W.S. Rasband, NIH, Bethesda, Maryland) imaging software. Timelapse imaging data from 4 adjacent quadrants of each well were montaged for scoring of CTL-target cell contacts. Instantaneous velocities for individual CTL were recorded using the MtrackJ plugin (E Meijering, Biomedical Imaging Group, University Medical Center Rotterdam, Netherlands) of ImageJ, and arrest coefficients were calculated as the percentage of time that a cell’s instantaneous velocity was <2 µm/min. Percentage of killed targets was calculated [% killed = (sytox^+^ targets at time 10 h – sytox^+^ targets at time 0)/sytox^−^ targets at time 0].

### Statistical Analysis

Data analysis was performed in Prism (Graphpad Software). p values were calculated using two-way ANOVA followed by a Bonferroni post-tests or with the Kruskal-Wallis nonparametric one-way ANOVA test followed by a Dunn’s post-test.

## Supporting Information

File S1 Contains Figure S1–S5. **Figure S1.** Related to Figure 1. *In situ* reporters enabling continuous videomicroscopy of CTLs and primary CD4^+^ target cells in ECM. (**A**) CTXB labeling does not impair A14 CTL killing of peptide-pulsed targets in a traditional ^51^Cr release assay in liquid culture. (**B**) Duration of individual CTL-target engagements was quantified for A14 CTLs and CD4^+^ T cells (without antigen) co-cultured in ECM and imaged by videomicroscopy. Dotted line indicates the median, with time in min (left axis) and hr (right). Bars indicate mean ± SEM. (**C**) CTL hit rate defined as the number of CD4^+^ T cell targets (without antigen) encountered by individual A14 CTLs per hour determined from videomicroscopy analysis of A14 CTLs co-cultured in ECM with antigen-free primary CD4^+^ T cells (E:T ratio 1∶2). Bars indicate mean ± SEM. **Figure S2**. Related to Figure 2. CTLs exhibit dynamic engagements with HIV-infected CD4^+^ target cells. CTL arrest coefficients (defined as % of time each CTL exhibited an instantaneous velocity of ≤2 µm/min) were determined for individual CTL-infected target engagements leading to HIV-infected target death or escape during hours 1–2 of imaging. Data shown are from 1 representative of 3 independent experiments. Bars indicate mean ± SEM. **Figure S3.** Related to Figure 4. Target cell motility directly impacts CTL function. (A) Time required to deliver a lethal hit is not altered by prior failed contacts of targets with CD8^+^ T cells. A14 CTLs were co-cultured in collagen with peptide-pulsed CD4^+^ target cells (20 nM SL9) for 10 hr. The duration of the ultimately lethal CTL contact is shown for targets killed by the first or *n*th A14 CTL encountered in the matrix. (**B**) The impact of target motility on CTL function is not limited to CD4^+^ T cell targets since a similar effect was observed for motile B cell targets. BCL target cells (controls or pulsed with KK10 gag peptide) were spun to the bottom of collagen matrices prior to gelation to allow binding to the underlying glass substrate, which was coated with anti-CD19 antibody and ICAM or ICAM alone. E501 CTLs were added to the matrix (E:T ratio 1∶2) and target/CTL dynamics were recorded by videomicroscopy for 10 hr. Engagement histories were recorded for motile or immobilized targets that were killed. **Figure S4.** Related to Figure 6. CTL exhibit a prolonged TCR-dependent, CTL-intrinsic migration arrest after successfully engaging and killing an initial target. (**A**) CTL arrest coefficients after 2, 4, or 9 hr of co-culture for A14 CTL engaging CD4^+^ T-cells (0, 0.1, 2, 20 nM SL9) in collagen (E:T ratio 1∶2, 10 hr timelapse). Data shown are from 1 representative of 5 independent experiments. Bars indicate mean ± SEM. (**B**) Elapsed time between initial target cell killing and subsequent target contacts for individual A14 CTL engaging CD4^+^ T-cells (20 nM SL9) in collagen (E:T ratio 1∶2, 10 hr timelapse). Data shown are from 1 representative of 5 independent experiments. Bars indicate mean ± SEM. (**C**) CTLs retain large stores of lytic enzymes at antigen doses eliciting significant CTL activity. E501 CTL were incubated with KK10 peptide-pulsed CD4 T cell targets in liquid culture for 6 hr and lytic granule content was analyzed by flow cytometry. Plots are gated on CD8^+^ cells. **Figure S5.** Related to Figure 7: CTLs rapidly transition from killing to sustained non-lytic effector secretion during prolonged arrest. A14 CTLs were co-cultured with antigen-pulsed CD4^+^ T cell targets in collagen (E:T ratio 1∶2) and imaged. The percentage of CTL engaged with a target was assessed at the indicated times.
(ZIP)

File S2 Contains videos S1–S7. **Video S1.** Related to Figure 1. Target cell motility within collagen gels vs convection within traditional liquid culture. CD4^+^ T cells were imaged within 3D ECM (left panel) and in liquid culture (right panel). Elapsed time shown in min:sec; scale bar 20 µm. **Video S2.** Related to Figure 1. CTLs and target cells migrate within 3D ECM and CTL killing is not observed in the absence of cognate antigen. A control culture containing primary HIV-specific CTL and autologous CD4^+^ T cells (not pulsed with any peptides) was imaged within 3D ECM. CTL were stained with CTXB (red) and targets were unlabeled. Dead cells are visualized with sytox green. Elapsed time shown in hr:min; scale bar 20 µm. **Video S3.** Related to Figure 1. A target cell is killed and permeabilized. The collagen matrix is infused with the membrane-impermeable dye sytox, which selectively fluoresces upon binding to DNA. Sytox permitted real-time objective identification of permeabilized cells *in situ*, as dead targets are marked by the rapid acquisition of green fluorescence. Shown is one representative example of an A14 CTL (indicated with red arrow) killing and permeabilizing a CD4^+^ T cell target (pulsed with 2 nM SL9 peptide, white arrow). Elapsed time is indicated in hours:min. Scale bar 20 µm. **Video S4.** Related to Figure 1. A target cell is killed and undergoes a “morphological death.” Not all CTL killing of target cells was accompanied by permeabilization. Non-permeabilizing kills were easily distinguished by morphological signs of death, which included blebbing of the target membrane and complete cessation of target movement. Shown is one representative example of an A14 CTL (CTXB, red) killing an unlabeled CD4^+^ T cell target (pulsed with 2 nM SL9 peptide). Elapsed time is indicated in hours:min. Scale bar 20 µm. **Videos S5–S8.** related to Figure 2. Recognition, engagement and killing of HIV-infected target cells in ECM. E501 CTLs were co-cultured in ECM with HLA-matched CD4^+^ T cell targets infected with NL4–3 HIV virus. Permeabilized targets are visualized *in situ* with sytox green. Videos S5, S7, and S8: target cells were from sorted, HIV-infected populations (>95% NL4-3-GFP^+^). Video S6 was acquired using an HIV-infected population of targets (∼30% p24^+^). The following cues are included: CTL, red arrow; target, white arrow; elapsed time shown in min or hr:min; scale bar 20 µm. **Video S5** A successful CTL-target engagement characteristic of a “direct hit kill.” **Video S6** A dramatic example of a “successful tether” followed by target death. **Video S7** An example of a target escape marked by a “failed tether.”
(ZIP)

File S3 Contains videos S8–S14. **Video S8** A failed CTL-target engagement characteristic of a “brush.” **Videos S9–S10.** Related to Figure 2 and 3. Primary HIV-specific CTL engage targets with dynamics similar to those observed with CTL clones. Primary, polyclonal HIV-specific CTL from subject 285873 were primed with targets bearing overlapping 18-mer peptide pools covering the full sequences of Gag, Pol, Nef, and Env. On day 14 the polyclonal CTL were labeled with CTXB and seeded in 3D ECM with autologous CD4^+^ T cell targets pulsed with the same peptide pools (100 ng/mL). Target permeabilization is visualized with sytox green. The following cues are included: CTL, red arrow; target, white arrow; scale bar 20 µm. Elapsed time shown in hr:min. **Video S9** Primary CTL engages a target and commits a direct hit kill. **Video S10** Primary CTL exhibits a failed tether, followed by target escape. **Videos S11–S12.** Related to Figure 4. Target cell motility impedes antigen recognition. A14 CTLs were double labeled with CTXB and the ratiometric calcium-sensing dye FURA-2AM prior to loading in ECM. A14 CTLs engaged HLA-matched CD4^+^ T cell targets pulsed with SL9 peptide (2 nM). The following video cues are included: On the x-axis CTL engagement with a live target is indicated in pink, prolonged CTL engagement with a killed target is indicated in gray; CTL, red arrow; target, white arrow; elapsed time shown in min; scale bar 20 µm. **Video S11** CTLs exhibit prolonged TCR signaling following a direct hit kill. The top video panel is a brightfield/CTXB fluorescence overlay of CTL-target engagement dynamics. TCR-dependent calcium signaling is represented in pseudocolor video (bottom video panel) and is quantitatively expressed over time as a normalized Fura ratio (right panel). **Video S12** CTLs exhibit weak TCR signaling during a failed tether. An A14 CTL engages but fails to kill an antigen-pulsed CD4^+^ T cell. The left video panel is a brightfield/CTXB fluorescence overlay of CTL-target engagement dynamics. TCR-dependent calcium signaling is represented in pseudocolor video (right panel) and is quantitatively expressed over time as a normalized Fura ratio (bottom panel). **Video S13.** Related to Figure 6. After a prolonged engagement with a killed target, CTL disengages and exhibits durable migration arrest. An A14 CTL (labeled with CTXB, red) was observed to kill an HLA-matched CD4^+^ T cell target pulsed with SL9 peptide (20 nM). The permeabilized target is marked with sytox green. Shown is time-lapse imaging revealing prolonged engagement with the killed target, followed by durable migration arrest after CTL disengagement. **Video S14.** Related to Figure 6. An A14 CTL engages, kills and permeabilizes an initial HLA-matched CD4^+^ T cell target. While still arrested and engaged with the initial killed target, the CTL catches and kills a subsequent target. After “serially killing” two targets, this CTL remained arrested and engaged with the killed targets, permitting escape of three additional targets observed to run over the CTL. Targets were pulsed with SL9 peptide (2 nM). Target permeabilization is visualized with sytox green.
(ZIP)
