# Supplementary figures and images for: High Avidity CD8+ T Cells Efficiently Eliminate Motile HIV-Infected Targets and Execute a Locally Focused Program of Anti-Viral Function
Source: PLoS One. 2014 Feb 13;9(2):e87873. doi: 10.1371/journal.pone.0087873 (PMC3923750; doi:10.1371/journal.pone.0087873)

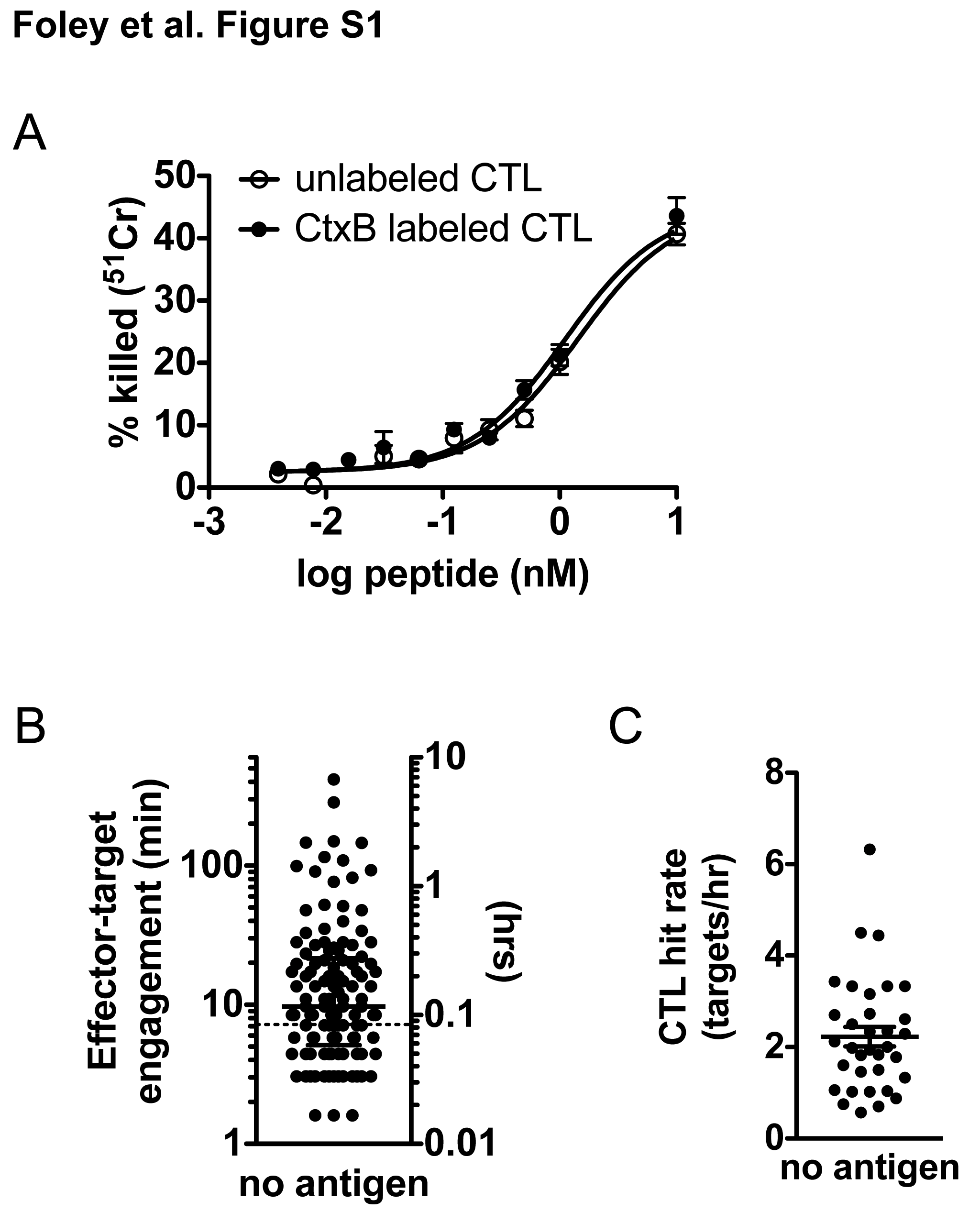

Supplement: File S1 — Contains Figure S1–S5. Figure S1. Related to Figure 1. In situ reporters enabling continuous videomicroscopy of CTLs and primary CD4+ target cells in ECM. (A) CTXB labeling does not impair A14 CTL killing of peptide-pulsed targets in a traditional 51Cr release assay in liquid culture. (B) Duration of individual CTL-target engagements was quantified for A14 CTLs and CD4+ T cells (without antigen) co-cultured in ECM and imaged by videomicroscopy. Dotted line indicates the median, with time in min (left axis) and hr (right). Bars indicate mean ± SEM. (C) CTL hit rate defined as the number of CD4+ T cell targets (without antigen) encountered by individual A14 CTLs per hour determined from videomicroscopy analysis of A14 CTLs co-cultured in ECM with antigen-free primary CD4+ T cells (E:T ratio 1∶2). Bars indicate mean ± SEM. Figure S2. Related to Figure 2. CTLs exhibit dynamic engagements with HIV-infected CD4+ target cells. CTL arrest coefficients (defined as % of time each CTL exhibited an instantaneous velocity of ≤2 µm/min) were determined for individual CTL-infected target engagements leading to HIV-infected target death or escape during hours 1–2 of imaging. Data shown are from 1 representative of 3 independent experiments. Bars indicate mean ± SEM. Figure S3. Related to Figure 4. Target cell motility directly impacts CTL function. (A) Time required to deliver a lethal hit is not altered by prior failed contacts of targets with CD8+ T cells. A14 CTLs were co-cultured in collagen with peptide-pulsed CD4+ target cells (20 nM SL9) for 10 hr. The duration of the ultimately lethal CTL contact is shown for targets killed by the first or nth A14 CTL encountered in the matrix. (B) The impact of target motility on CTL function is not limited to CD4+ T cell targets since a similar effect was observed for motile B cell targets. BCL target cells (controls or pulsed with KK10 gag peptide) were spun to the bottom of collagen matrices prior to gelation to allow binding to the und [file pone.0087873.s001.zip › Supplemental Figures_final/PLOSOne figures_ver 3_Figure S1.tif]

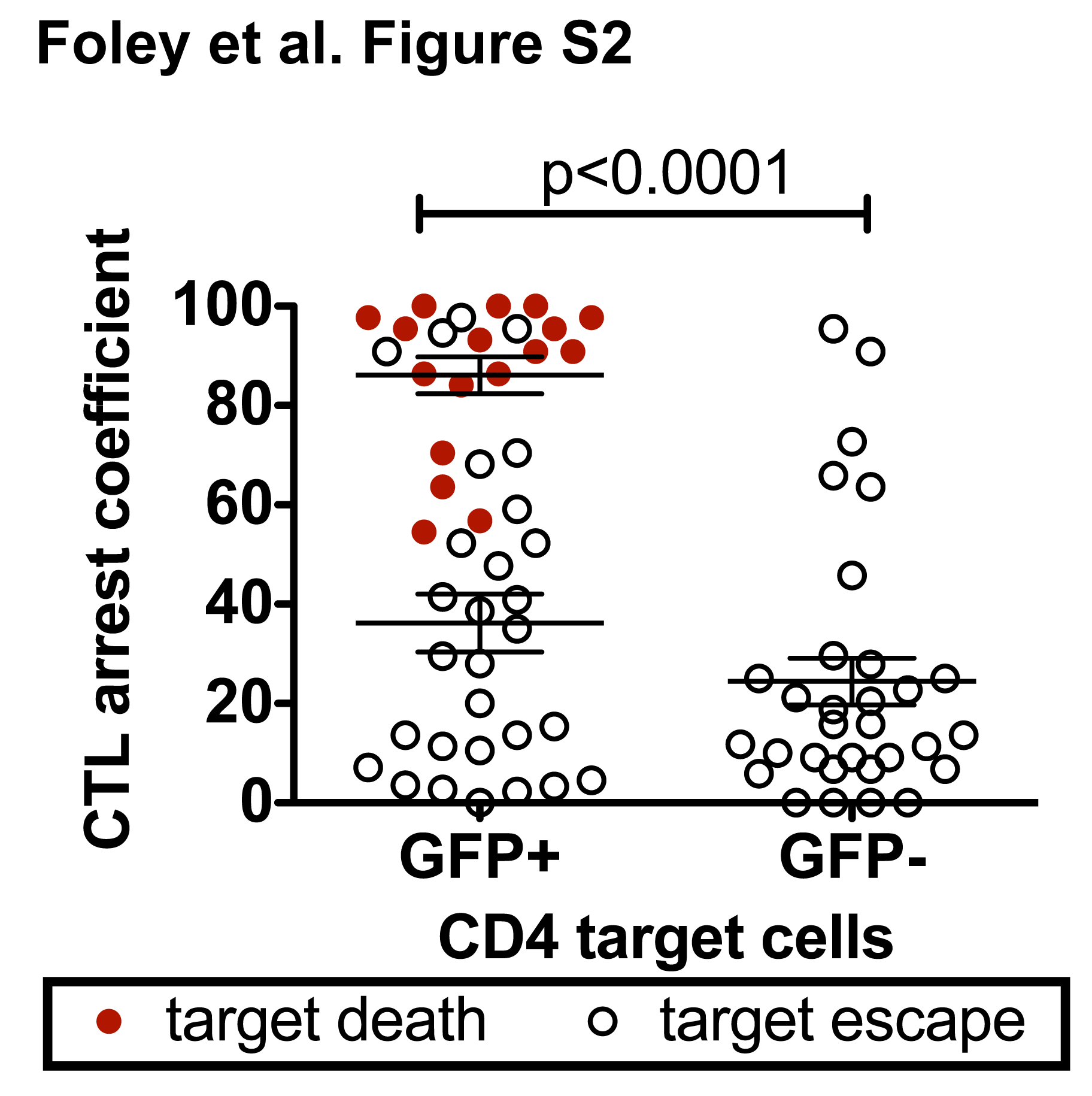

Supplement: File S1 — Contains Figure S1–S5. Figure S1. Related to Figure 1. In situ reporters enabling continuous videomicroscopy of CTLs and primary CD4+ target cells in ECM. (A) CTXB labeling does not impair A14 CTL killing of peptide-pulsed targets in a traditional 51Cr release assay in liquid culture. (B) Duration of individual CTL-target engagements was quantified for A14 CTLs and CD4+ T cells (without antigen) co-cultured in ECM and imaged by videomicroscopy. Dotted line indicates the median, with time in min (left axis) and hr (right). Bars indicate mean ± SEM. (C) CTL hit rate defined as the number of CD4+ T cell targets (without antigen) encountered by individual A14 CTLs per hour determined from videomicroscopy analysis of A14 CTLs co-cultured in ECM with antigen-free primary CD4+ T cells (E:T ratio 1∶2). Bars indicate mean ± SEM. Figure S2. Related to Figure 2. CTLs exhibit dynamic engagements with HIV-infected CD4+ target cells. CTL arrest coefficients (defined as % of time each CTL exhibited an instantaneous velocity of ≤2 µm/min) were determined for individual CTL-infected target engagements leading to HIV-infected target death or escape during hours 1–2 of imaging. Data shown are from 1 representative of 3 independent experiments. Bars indicate mean ± SEM. Figure S3. Related to Figure 4. Target cell motility directly impacts CTL function. (A) Time required to deliver a lethal hit is not altered by prior failed contacts of targets with CD8+ T cells. A14 CTLs were co-cultured in collagen with peptide-pulsed CD4+ target cells (20 nM SL9) for 10 hr. The duration of the ultimately lethal CTL contact is shown for targets killed by the first or nth A14 CTL encountered in the matrix. (B) The impact of target motility on CTL function is not limited to CD4+ T cell targets since a similar effect was observed for motile B cell targets. BCL target cells (controls or pulsed with KK10 gag peptide) were spun to the bottom of collagen matrices prior to gelation to allow binding to the und [file pone.0087873.s001.zip › Supplemental Figures_final/PLOSOne figures_ver 3_Figure S2.tif]

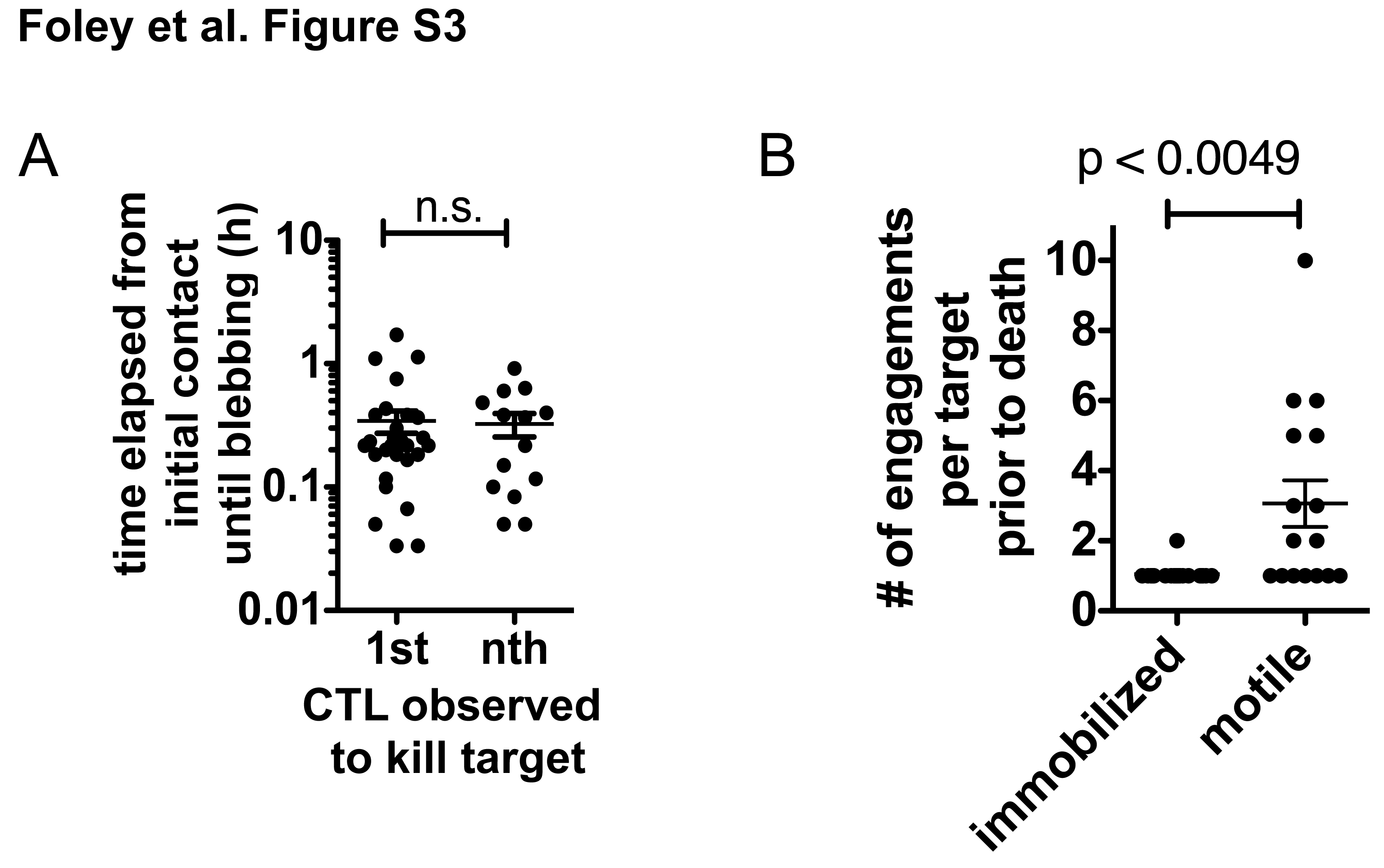

Supplement: File S1 — Contains Figure S1–S5. Figure S1. Related to Figure 1. In situ reporters enabling continuous videomicroscopy of CTLs and primary CD4+ target cells in ECM. (A) CTXB labeling does not impair A14 CTL killing of peptide-pulsed targets in a traditional 51Cr release assay in liquid culture. (B) Duration of individual CTL-target engagements was quantified for A14 CTLs and CD4+ T cells (without antigen) co-cultured in ECM and imaged by videomicroscopy. Dotted line indicates the median, with time in min (left axis) and hr (right). Bars indicate mean ± SEM. (C) CTL hit rate defined as the number of CD4+ T cell targets (without antigen) encountered by individual A14 CTLs per hour determined from videomicroscopy analysis of A14 CTLs co-cultured in ECM with antigen-free primary CD4+ T cells (E:T ratio 1∶2). Bars indicate mean ± SEM. Figure S2. Related to Figure 2. CTLs exhibit dynamic engagements with HIV-infected CD4+ target cells. CTL arrest coefficients (defined as % of time each CTL exhibited an instantaneous velocity of ≤2 µm/min) were determined for individual CTL-infected target engagements leading to HIV-infected target death or escape during hours 1–2 of imaging. Data shown are from 1 representative of 3 independent experiments. Bars indicate mean ± SEM. Figure S3. Related to Figure 4. Target cell motility directly impacts CTL function. (A) Time required to deliver a lethal hit is not altered by prior failed contacts of targets with CD8+ T cells. A14 CTLs were co-cultured in collagen with peptide-pulsed CD4+ target cells (20 nM SL9) for 10 hr. The duration of the ultimately lethal CTL contact is shown for targets killed by the first or nth A14 CTL encountered in the matrix. (B) The impact of target motility on CTL function is not limited to CD4+ T cell targets since a similar effect was observed for motile B cell targets. BCL target cells (controls or pulsed with KK10 gag peptide) were spun to the bottom of collagen matrices prior to gelation to allow binding to the und [file pone.0087873.s001.zip › Supplemental Figures_final/PLOSOne figures_ver 3_Figure S3.tif]

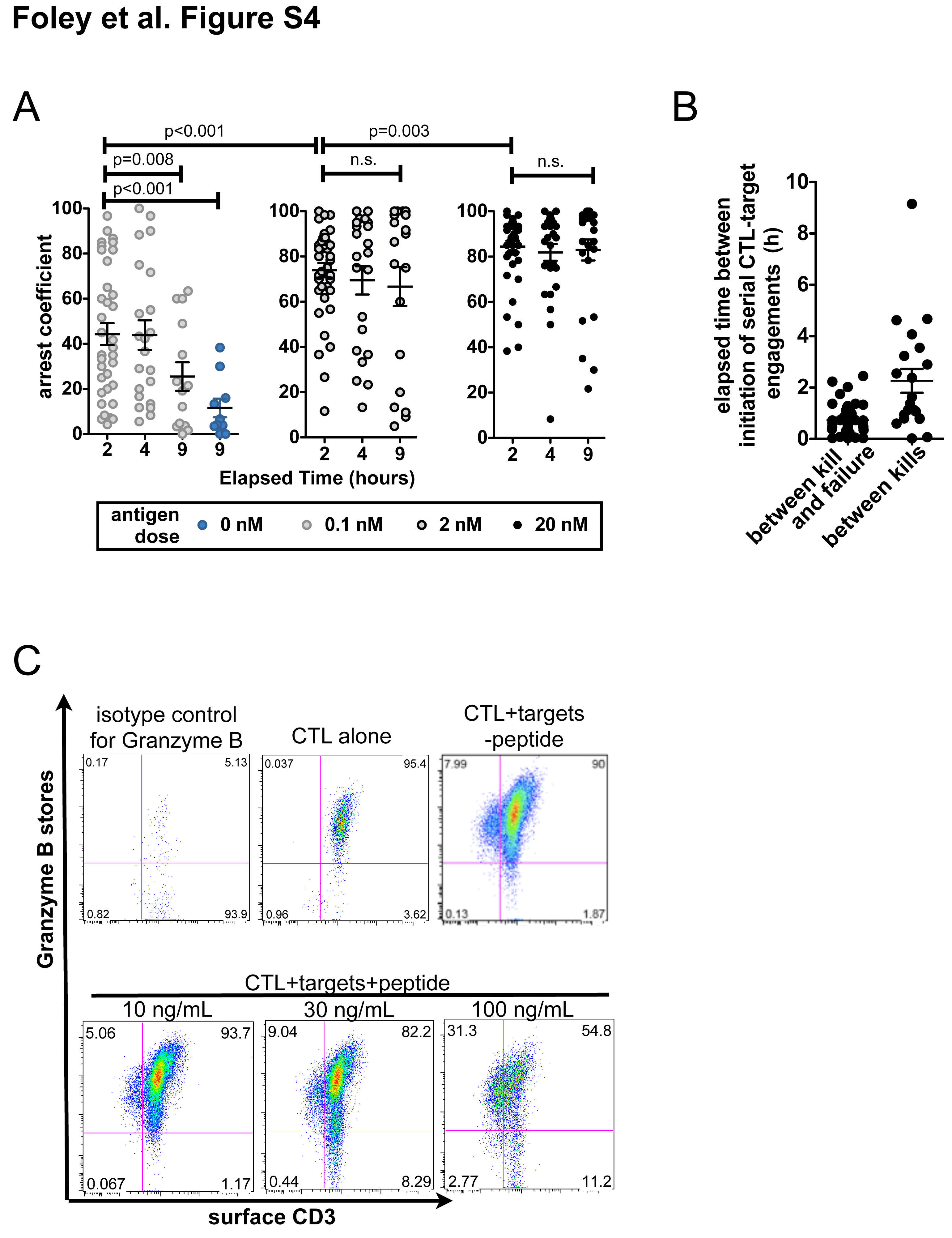

Supplement: File S1 — Contains Figure S1–S5. Figure S1. Related to Figure 1. In situ reporters enabling continuous videomicroscopy of CTLs and primary CD4+ target cells in ECM. (A) CTXB labeling does not impair A14 CTL killing of peptide-pulsed targets in a traditional 51Cr release assay in liquid culture. (B) Duration of individual CTL-target engagements was quantified for A14 CTLs and CD4+ T cells (without antigen) co-cultured in ECM and imaged by videomicroscopy. Dotted line indicates the median, with time in min (left axis) and hr (right). Bars indicate mean ± SEM. (C) CTL hit rate defined as the number of CD4+ T cell targets (without antigen) encountered by individual A14 CTLs per hour determined from videomicroscopy analysis of A14 CTLs co-cultured in ECM with antigen-free primary CD4+ T cells (E:T ratio 1∶2). Bars indicate mean ± SEM. Figure S2. Related to Figure 2. CTLs exhibit dynamic engagements with HIV-infected CD4+ target cells. CTL arrest coefficients (defined as % of time each CTL exhibited an instantaneous velocity of ≤2 µm/min) were determined for individual CTL-infected target engagements leading to HIV-infected target death or escape during hours 1–2 of imaging. Data shown are from 1 representative of 3 independent experiments. Bars indicate mean ± SEM. Figure S3. Related to Figure 4. Target cell motility directly impacts CTL function. (A) Time required to deliver a lethal hit is not altered by prior failed contacts of targets with CD8+ T cells. A14 CTLs were co-cultured in collagen with peptide-pulsed CD4+ target cells (20 nM SL9) for 10 hr. The duration of the ultimately lethal CTL contact is shown for targets killed by the first or nth A14 CTL encountered in the matrix. (B) The impact of target motility on CTL function is not limited to CD4+ T cell targets since a similar effect was observed for motile B cell targets. BCL target cells (controls or pulsed with KK10 gag peptide) were spun to the bottom of collagen matrices prior to gelation to allow binding to the und [file pone.0087873.s001.zip › Supplemental Figures_final/PLOSOne figures_ver 3_Figure S4.tif]

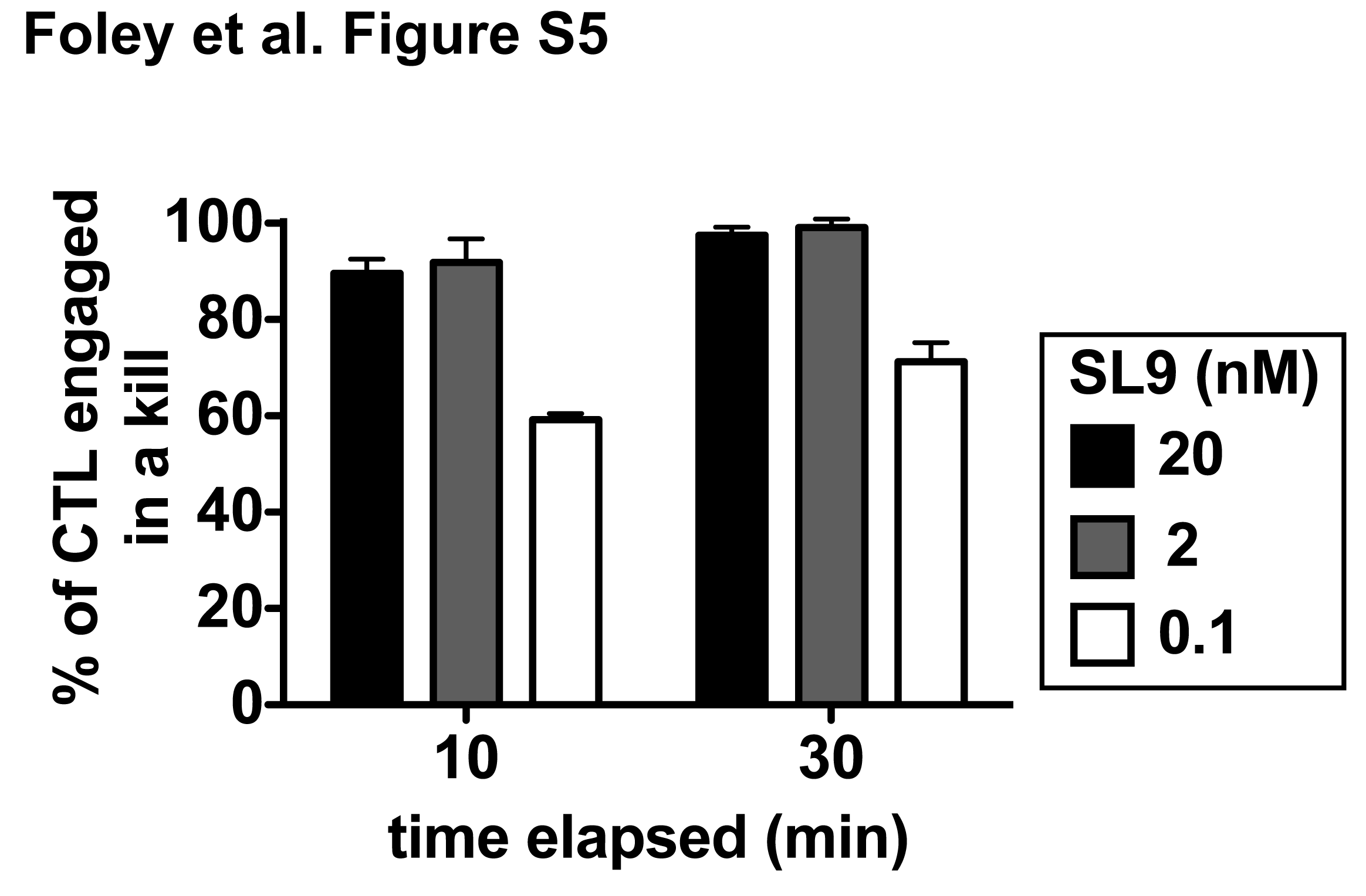

Supplement: File S1 — Contains Figure S1–S5. Figure S1. Related to Figure 1. In situ reporters enabling continuous videomicroscopy of CTLs and primary CD4+ target cells in ECM. (A) CTXB labeling does not impair A14 CTL killing of peptide-pulsed targets in a traditional 51Cr release assay in liquid culture. (B) Duration of individual CTL-target engagements was quantified for A14 CTLs and CD4+ T cells (without antigen) co-cultured in ECM and imaged by videomicroscopy. Dotted line indicates the median, with time in min (left axis) and hr (right). Bars indicate mean ± SEM. (C) CTL hit rate defined as the number of CD4+ T cell targets (without antigen) encountered by individual A14 CTLs per hour determined from videomicroscopy analysis of A14 CTLs co-cultured in ECM with antigen-free primary CD4+ T cells (E:T ratio 1∶2). Bars indicate mean ± SEM. Figure S2. Related to Figure 2. CTLs exhibit dynamic engagements with HIV-infected CD4+ target cells. CTL arrest coefficients (defined as % of time each CTL exhibited an instantaneous velocity of ≤2 µm/min) were determined for individual CTL-infected target engagements leading to HIV-infected target death or escape during hours 1–2 of imaging. Data shown are from 1 representative of 3 independent experiments. Bars indicate mean ± SEM. Figure S3. Related to Figure 4. Target cell motility directly impacts CTL function. (A) Time required to deliver a lethal hit is not altered by prior failed contacts of targets with CD8+ T cells. A14 CTLs were co-cultured in collagen with peptide-pulsed CD4+ target cells (20 nM SL9) for 10 hr. The duration of the ultimately lethal CTL contact is shown for targets killed by the first or nth A14 CTL encountered in the matrix. (B) The impact of target motility on CTL function is not limited to CD4+ T cell targets since a similar effect was observed for motile B cell targets. BCL target cells (controls or pulsed with KK10 gag peptide) were spun to the bottom of collagen matrices prior to gelation to allow binding to the und [file pone.0087873.s001.zip › Supplemental Figures_final/PLOSOne figures_ver 3_Figure S5.tif]
